# Supplementary material for: Fine-Scaled Selection of Resting and Hunting Habitat by Leopard Cats (Prionailurus bengalensis) in a Rural Human-Dominated Landscape in Taiwan
Source: Animals (Basel). 2023 Jan 8;13(2):234. doi: 10.3390/ani13020234 (PMC9854813; doi:10.3390/ani13020234)

Fine-scaled selection of resting and hunting habitat by leopard cats (*Prionailurus bengalensis*) in a rural human-dominated landscape in Taiwan

Esther van der Meer[1 ID], Hans Dullemont[1], Ching-Hao Wang[1], Jun-Wei Zhang[1], Jun-Liang Lin[2], Kurtis Jai-Chyi Pei[1], Yu-Ching Lai[2*]

[1]Institute of Wildlife Conservation, College of Veterinary Medicine, National Pingtung University of Science and Technology, Pingtung, Taiwan
[2]Department of Landscape Architecture and Environmental Design, Huafan University, New Taipei City, Taiwan

Table S1. Characteristics of the studied leopard cats: sex (M = male, F = female), age, body weight and body score of the studied leopard cats, start and end date of radio-tracking, number of days followed, number of night sessions and reason for termination of the study. Body score is measured on a scale of 1-5, a score of 2.5-3 is considered healthy for domestic cats (Baldwin *et al.*, 2020).

ID	Sex	Age	Body weight (kg)	Body score	Start tracking	End tracking	Number of days followed	Number of night sessions	Reason termination study
MZM01	M	Ad (1-2 yrs.)	4.40	2	15-5-2019	20-6-2019	38	2	Mortality (cause unknown)
MZM02	M	SA (7-8 months)	2.93	2	3-6-2019	21-8-2019	81	7	Mortality (roadkill)
MZF03	F	SA (5-6 months)	1.50	3	24-7-2019	27-7-2019	4	0	Mortality (domestic dogs)
MZF04[1]	F	Ad (2-3 yrs.)	2.84	2	22-8-2019	8-11-2020	445	15	Collar battery drained
MZM05	M	Ad (2-3 yrs.)	3.40	2	5-10-2019	16-12-2019	73	5	Mortality (domestic dogs)
MZF06[2]	F	Ad (3-4 yrs.)	2.68	2	17-3-2020	4-6- 2021	445	11	Collar battery drained
MZM07	M	Ad (2-3 yrs.)	3.77	2-2.5	6-4-2020	1-4-2021	361	7	Collar drop-off
MZM08	M	Ad (4-5 yrs.)	4.00	3-3.5	11-5-2020	17-7-2021	433	8	Collar battery drained

[1]Maternal period - 2019: No litter, 2020: 23rd of July-8th of November (study terminated prior to dispersal kittens)
[2]Maternal period - 2020: 16th of July-2nd of August (litter failed), 2021: 3rd of April-4th of June (study terminated prior to dispersal kittens)

Baldwin, K.; Bartgens, J.; Buffington, J.; Freeman, L.M.; Grabow, M.; Legred, J.; Ostwald Jr, D. AAHA nutritional assessment guidelines for dogs and cats. *J. Am. Anim. Hosp. Assoc.* **2010**, *46*, 285–296. Available online: https://www.aaha.org/globalassets/02-guidelines/nutritional-assessment/nutritionalassessmentguidelines.pdf (accessed 21 December 2022)

Table S2. Mean proportions of vegetation and other ground cover types for land use subclasses at leopard cat daytime resting sites and random points, only including vegetation and other ground cover types for which total sample size of random points and/or resting sites was > 5.

			Trees	Shrub	Bamboo	Reed	Grass	Herbs	Stones	Bare	River blocks	Building	Tar road	Dirt road	Other
Abandoned	All	Mean	0.24	0.07	0.02	0.18	0.04	0.42	0	0.04	0	0	0	0	0
orchard	(*n* = 80)	SE	0.02	0.01	0.01	0.02	0.02	0.03	0	0.01	0	0	0	0	0
	Random	Mean	0.23	0.01	0.01	0.08	0.17	0.48	0	0.02	0	0	0	0	0
	(*n* = 15)	SE	0.06	0.01	0.01	0.04	0.08	0.09	0	0.01	0	0	0	0	0
	Rest	Mean	0.24	0.08	0.02	0.20	0.01	0.40	0	0.04	0	0	0	0	0
	(*n* = 65)	SE	0.02	0.02	0.01	0.02	0.01	0.03	0	0.01	0	0	0	0	0
Arable	All	Mean	0.03	0.03	0	0.01	0.02	0.27	0	0.55	0	0.03	0	0	0.06
	(*n* = 23)	SE	0.02	0.02	0	0.01	0.01	0.07	0	0.08	0	0.03	0	0	0.04
	Random	Mean	0.02	0.03	0	0.01	0.02	0.26	0	0.60	0	0	0	0	0.06
	(*n* = 21)	SE	0.01	0.02	0	0.01	0.01	0.07	0	0.08	0	0	0	0	0.05
	Rest	Mean	0.18	0	0	0	0	0.38	0	0	0	0.40	0	0	0.05
	(*n* = 2)	SE	0.08	0	0	0	0	0.38	0	0	0	0.40	0	0	0.05
Orchard	All	Mean	0.26	0.01	0	0.01	0.04	0.63	0	0.06	0	0	0	0	0
	(*n* = 255)	SE	0.01	0	0	0	0.01	0.01	0	0.01	0	0	0	0	0
	Random	Mean	0.26	0	0	0	0.03	0.64	0	0.06	0	0	0	0	0
	(*n* = 208)	SE	0	0	0	0	0.01	0.01	0	0.01	0	0	0	0	0
	Rest	Mean	0.26	0.03	0	0.03	0.08	0.57	0	0.03	0	0	0	0	0
	(*n* = 47)	SE	0.02	0.02	0	0.02	0.03	0.04	0	0.01	0	0	0	0	0
Pine	All	Mean	0.38	0.08	0.01	0.17	0	0.29	0	0.08	0	0	0	0	0
	(*n* = 8)	SE	0.05	0.04	0.01	0.07	0	0.10	0	0.05	0	0	0	0	0
	Random	Mean	0.50	0	0	0	0	0.30	0	0.20	0	0	0	0	0
	(*n* = 1)	SE	-	-	-	-	-	-	-	-	-	-	-	-	-
	Rest	Mean	0.36	0.09	0.01	0.19	0	0.29	0	0.06	0	0	0	0	0
	(*n* = 7)	SE	0.05	0.05	0.01	0.07	0	0.11	0	0.06	0	0	0	0	0
Bamboo	All	Mean	0.01	0.01	0.57	0.02	0	0.09	0	0.30	0	0	0	0	0
	(*n* = 9)	SE	0.01	0.01	0.04	0.02	0	0.02	0	0.06	0	0	0	0	0

	Random	Mean	0	0.02	0.63	0	0	0.08	0	0.27	0	0	0	0	0
	(n = 3)	SE	0	0.02	0.09	0	0	0.02	0	0.09	0	0	0	0	0
	Rest	Mean	0.01	0.01	0.54	0.03	0	0.09	0	0.32	0	0	0	0	0
	(n = 6)	SE	0.01	0.01	0.05	0.02	0	0.02	0	0.07	0	0	0	0	0
Forest	All	Mean	0.25	0.11	0.10	0.19	0	0.27	0.01	0.07	0	0	0	0	0
	(n = 882)	SE	0.01	0	0	0.01	0	0.01	0	0	0	0	0	0	0
	Random	Mean	0.26	0.09	0.13	0.12	0	0.28	0	0.11	0	0	0	0	0
	(n = 264)	SE	0.01	0.01	0.01	0.01	0	0.01	0	0.01	0	0	0	0	0
	Rest	Mean	0.24	0.12	0.09	0.22	0.01	0.26	0.01	0.06	0	0	0	0	0
	(n = 618)	SE	0.01	0	0.01	0.01	0	0.01	0	0	0	0	0	0	0
Riverbed	All	Mean	0.01	0.02	0	0.28	0.06	0.08	0.11	0.44	0	0	0	0	0
	(n = 77)	SE	0	0.01	0	0.04	0.02	0.02	0.03	0.05	0	0	0	0	0
	Random	Mean	0	0	0	0.11	0.03	0.03	0.14	0.69	0	0	0	0	0
	(n = 44)	SE	0	0	0	0.04	0.01	0.01	0.04	0.06	0	0	0	0	0
	Rest	Mean	0.03	0.05	0	0.49	0.09	0.15	0.07	0.12	0	0	0	0	0
	(n = 33)	SE	0.01	0.01	0	0.06	0.04	0.03	0.03	0.05	0	0	0	0	0
Riverine habitat	All	Mean	0.17	0.04	0	0.51	0.09	0.10	0.04	0.04	0.01	0	0	0	0
	(n = 648)	SE	0.01	0	0	0.01	0.01	0.01	0	0	0	0	0	0	0
	Random	Mean	0.13	0.02	0	0.68	0.04	0.06	0.01	0.06	0	0	0	0	0
	(n = 108)	SE	0.02	0.01	0	0.03	0.01	0.01	0	0.01	0	0	0	0	0
	Rest	Mean	0.18	0.04	0	0.48	0.10	0.11	0.05	0.03	0.01	0	0	0	0
	(n = 540)	SE	0.01	0	0	0.01	0.01	0.01	0	0	0	0	0	0	0
Unused land	All	Mean	0.12	0.07	0.02	0.45	0.01	0.28	0.01	0.03	0	0	0	0	0
	(n = 275)	SE	0.01	0.01	0	0.02	0	0.01	0	0.01	0	0	0	0	0
	Random	Mean	0.15	0.02	0.04	0.24	0.02	0.40	0	0.13	0	0	0	0	0
	(n = 38)	SE	0.03	0.01	0.02	0.05	0.02	0.05	0	0.04	0	0	0	0	0
	Rest	Mean	0.12	0.08	0.01	0.49	0.01	0.26	0.01	0.02	0	0	0	0	0
	(n = 237)	SE	0.01	0.01	0	0.02	0	0.01	0	0	0	0	0	0	0

Table S3. Overview of occurrence and abundance of the leopard cat's main prey species at leopard cat trap locations at the interface of natural habitat and orchards in the farmland, and natural habitat along the Da'an river.

		**Farmland[1]**		**River[2]**	
	**Species**	**Percentage of traps visited**	**Visits/100 trap nights**	**Percentage of traps visited**	**Visits/100 trap nights**
Murids	Shrews	0	0	10.0	0.7
	Mice	20.0	0.3	70.0	14.3
	Rats	40.0	1.2	20.0	2.1
	**Total**	**60.0**	**1.6**	**100**	**17.0**
Other small mammals	Hares	0	0	10.0	0.1
	Squirrels	20.0	0.2	0	0
	**Total**	**20.0**	**0.2**	**10.0**	**0.1**
Birds	Doves	66.7	6.7	40.0	8.3
	Hwameis	33.3	8.8	30.0	7.4
	Magpies	26.7	0.6	20.0	0.3
	Partridges	33.3	1.4	20.0	1.6
	Pheasants	20.0	0.2	0	0
	Quails	0	0	40.0	5.0
	Small birds other	66.7	4.0	80.0	5.3
	**Total**	**86.7**	**21.7**	**100**	**27.9**

[1]Number of traps in the farmland was 15, number of trap nights 1857 (Van der Meer *et al.*, 2022)

[2]Number of traps along the river was 10, number of trap nights 1050 (Van der Meer *et al.*, 2022)

Van der Meer, E.; Dullemont, H.; Chen, W.L.; Chang, A.M.; Chen, C.C.; Pei, K.J.C; Lai, Y.C. Live capture and handling of Taiwanese leopard cats *(Prionailurus bengalensis)*: an evaluation of trap designs and capture protocols. *Wildl. Biol.* **2022**, *4*, e01032. doi: 10.1002/wlb3.01032.

Table S4. Mean proportions of vegetation and other ground cover types in the riverbed and riverine habitat of the Da'an river in which the river leopard cats reside versus the rivers in the mountainous agricultural area in which the mountain leopard cats reside at all locations (random + resting), random points and daytime resting sites (Rest).

			Trees	Shrub	Bamboo	Reed	Grass	Herbs	Stones	Bare	River blocks	Building	Tar road	Dirt road	Other
Riverbed Da'an river	All	Mean	0	0	0	0.10	0.06	0.04	0.16	0.64	0	0	0	0	0
	(*n* = 51)	SE	0	0	0	0.03	0.02	0.01	0.04	0.06	0	0	0	0	0
	Random	Mean	0	0	0	0.10	0.03	0.01	0.15	0.70	0	0	0	0	0
	(*n* = 41)	SE	0	0	0	0.04	0.02	0.01	0.05	0.06	0	0	0	0	0
	Rest	Mean	0.01	0.02	0	0.10	0.20	0.14	0.18	0.36	0	0	0	0	0
	(*n* = 10)	SE	0.01	0.02	0	0.05	0.07	0.05	0.10	0.14	0	0	0	0	0
Riverbed mountainous agricultural area	All	Mean	0.03	0.05	0	0.63	0.04	0.16	0.02	0.08	0	0	0	0	0
	(*n* = 26)	SE	0.01	0.02	0	0.05	0.04	0.03	0.01	0.04	0	0	0	0	0
	Random	Mean	0	0	0	0.30	0	0.17	0	0.53	0	0	0	0	0
	(*n* = 3)	SE	0	0	0	0.25	0	0.09	0	0.17	0	0	0	0	0
	Rest	Mean	0.04	0.06	0	0.67	0.04	0.16	0.02	0.02	0	0	0	0	0
	(*n* = 23)	SE	0.01	0.02	0	0.05	0.04	0.03	0.01	0.02	0	0	0	0	0
Riverine Da'an river	All	Mean	0.18	0.04	0	0.51	0.09	0.10	0.04	0.04	0.01	0	0	0	0
	(*n* = 643)	SE	0.01	0	0	0.01	0.01	0.01	0	0	0	0	0	0	0
	Random	Mean	0.13	0.02	0	0.68	0.03	0.06	0.01	0.06	0	0	0	0	0
	(*n* = 107)	SE	0.02	0.01	0	0.03	0.01	0.01	0	0.01	0	0	0	0	0
	Rest	Mean	0.18	0.04	0	0.48	0.10	0.10	0.05	0.03	0.01	0	0	0	0
	(*n* = 536)	SE	0.01	0	0	0.01	0.01	0.01	0	0	0	0	0	0	0
Riverine habitat mountainous agricultural area	All	Mean	0	0.02	0	0.62	0.14	0.22	0	0	0	0	0	0	0
	(*n* = 5)	SE	0	0.02	0	0.12	0.14	0.10	0	0	0	0	0	0	0
	Random	Mean	0	0	0	0.30	0.70	0	0	0	0	0	0	0	0
	(*n* = 1)	SE	-	-	-	-	-	-	-	-	-	-	-	-	-
	Rest	Mean	0	0.03	0	0.70	0	0.28	0	0	0	0	0	0	0
	(*n* = 4)	SE	0	0.03	0	0.11	0	0.11	0	0	0	0	0	0	0

Figure S1. Examples of leopard cat daytime resting sites within land use subclasses commonly selected by the studied individuals (n ≥ 3): (a) Riverine habitat, (b) Unused land, (c) Forest, (d) Riverbed and (e) Abandoned orchard (*photo credit:* Esther van der Meer).

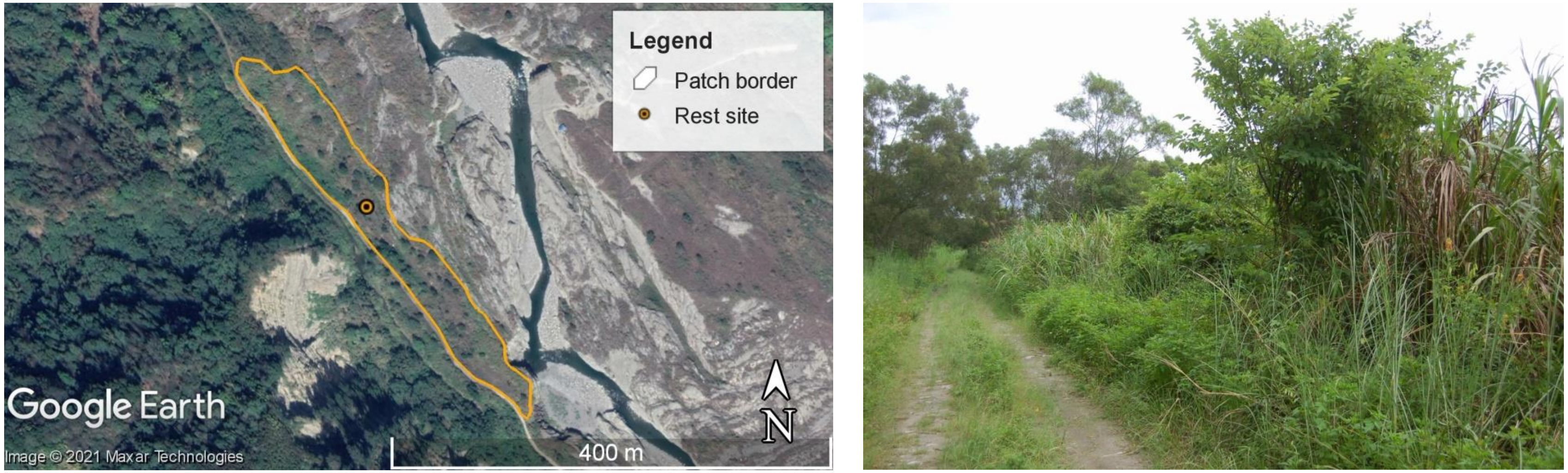

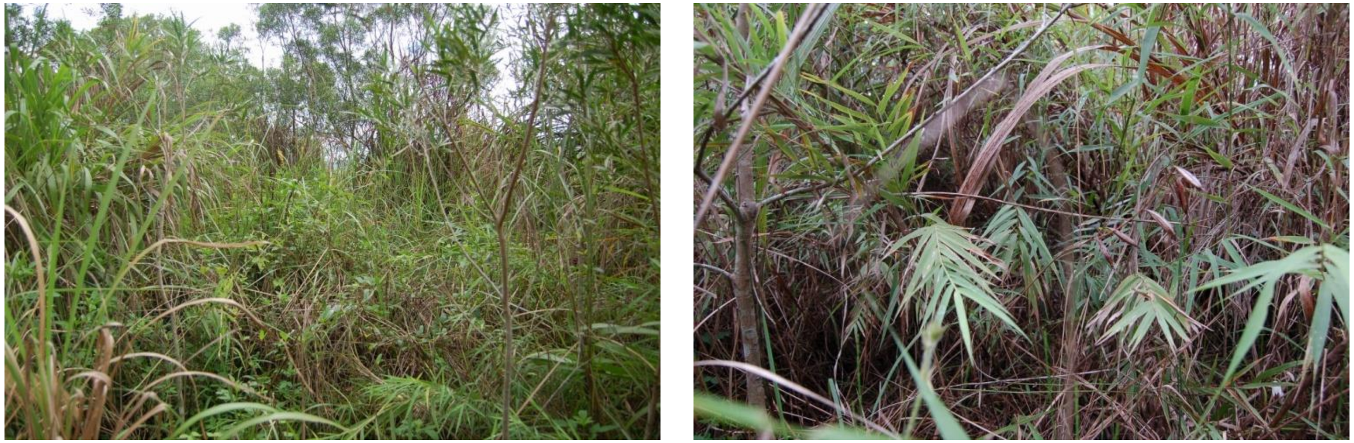

(a) Riverine habitat

From left to right:
Location
Street view
Vegetation
Ground cover

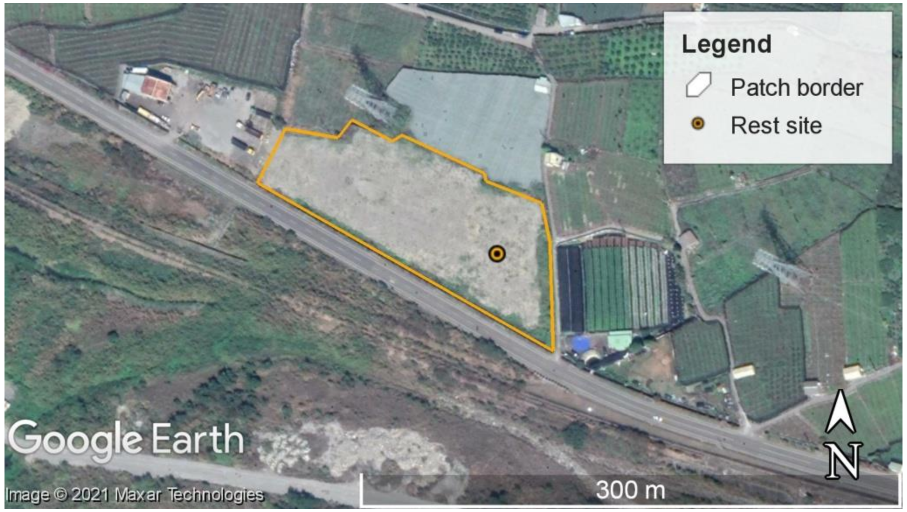

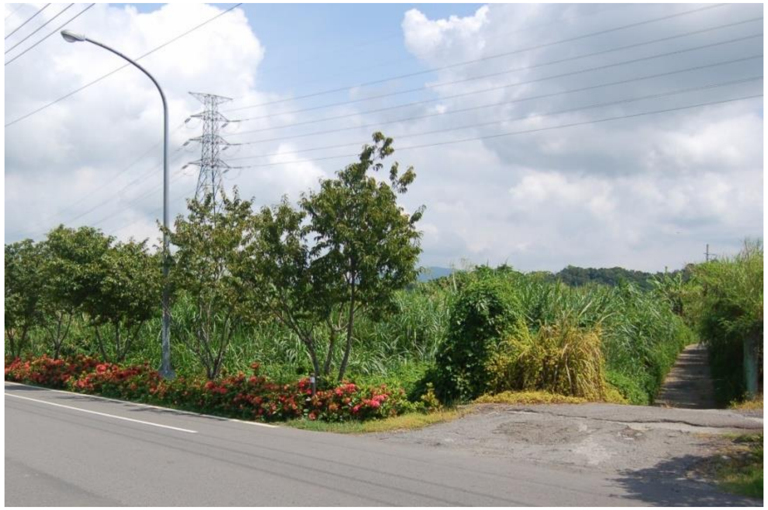

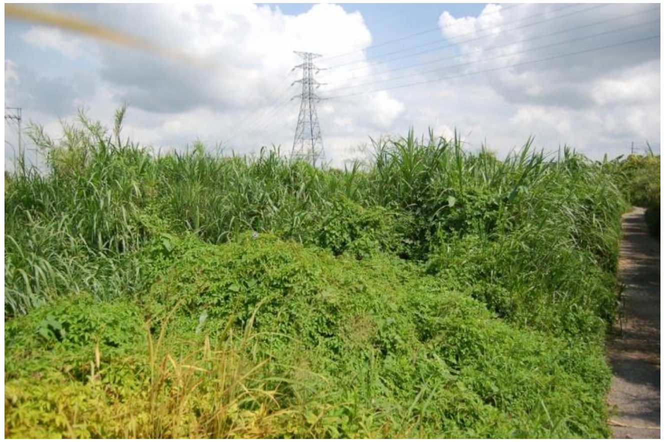

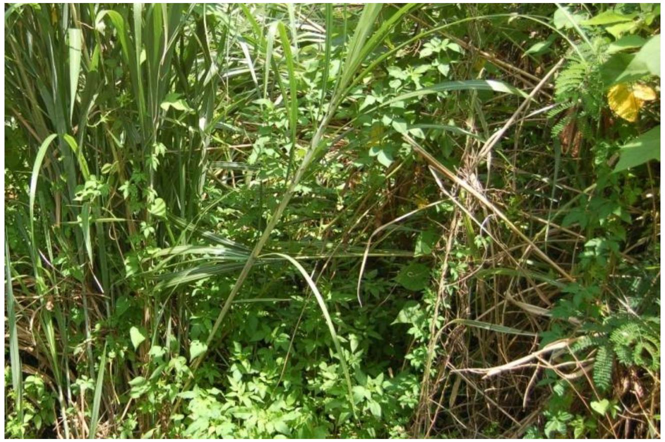

(b) Unused land

From left to right:
Location
Street view
Vegetation
Ground cover

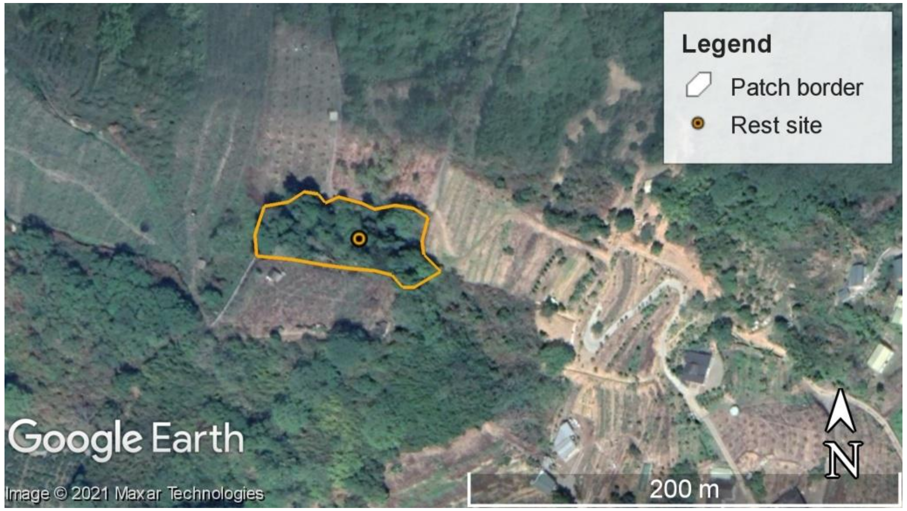

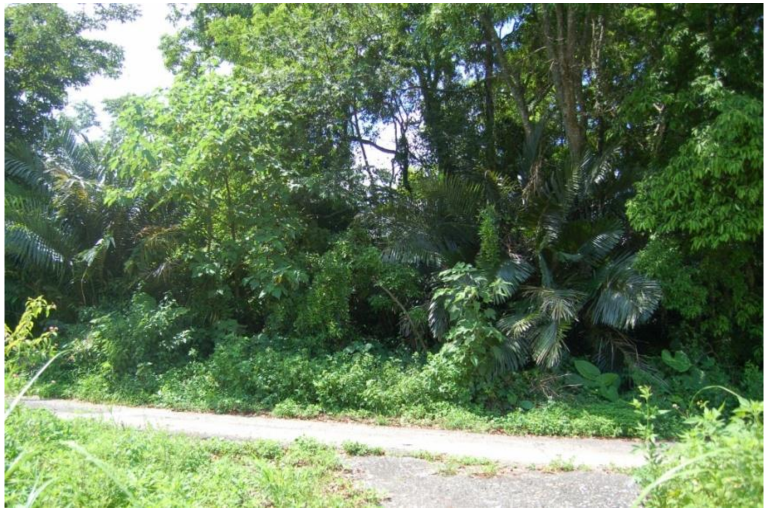
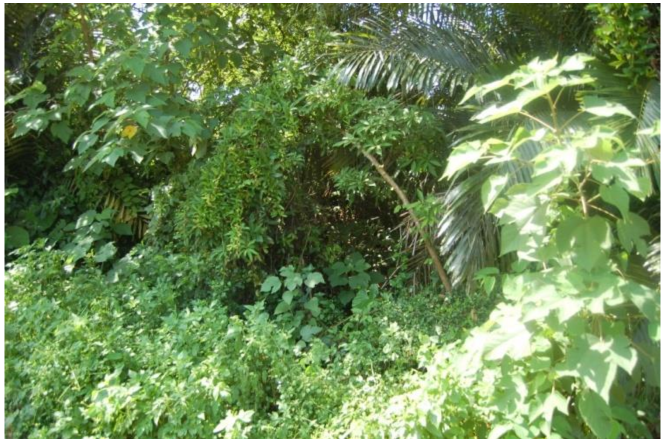
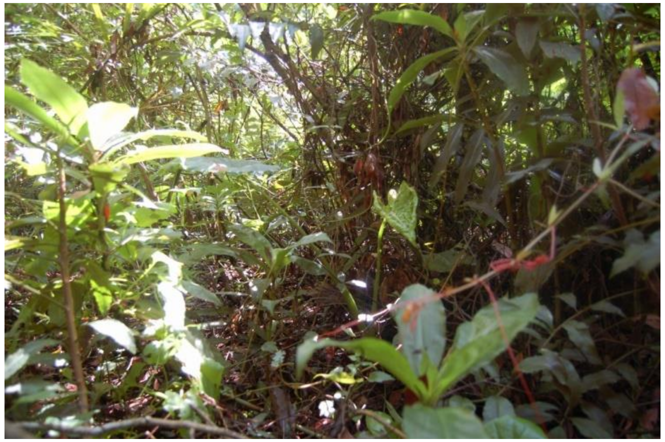

(c) Forest

From left to right:
Location
Street view
Vegetation
Ground cover

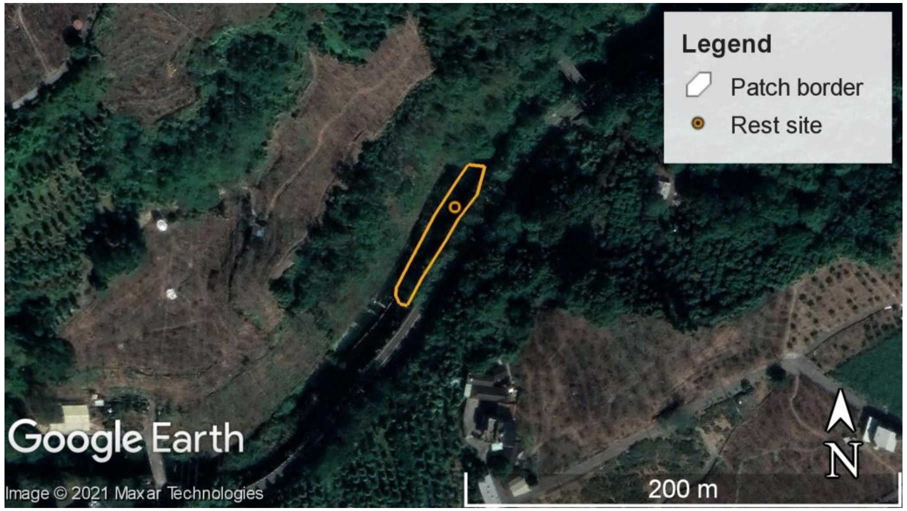

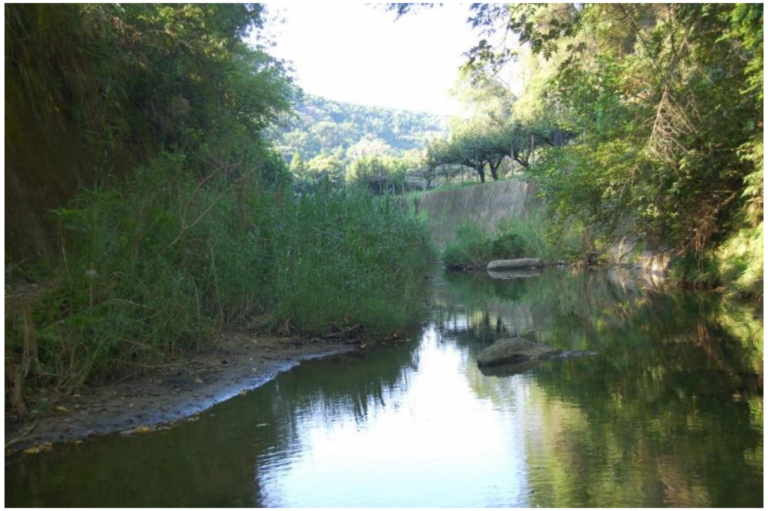
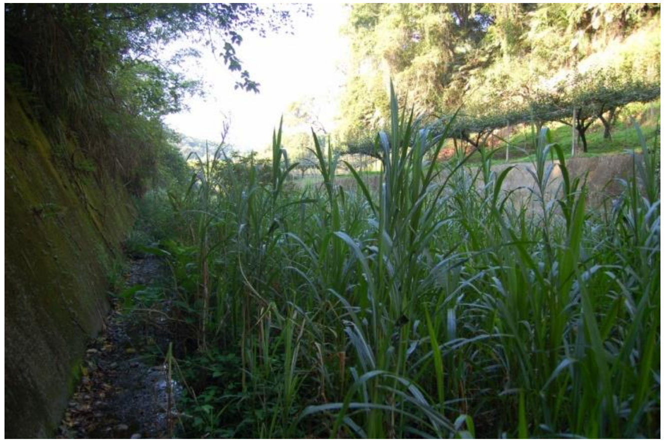
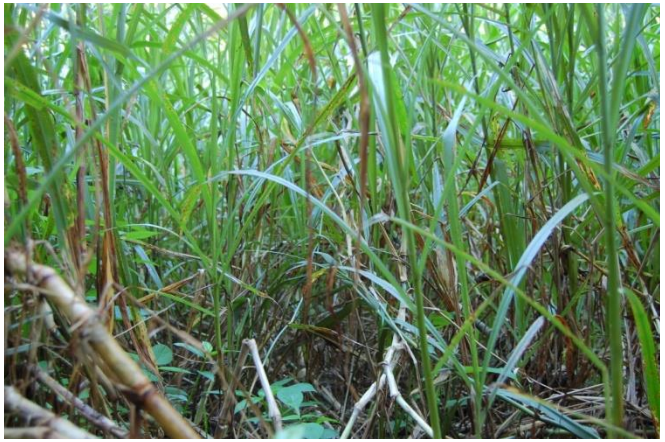

(d) Riverbed

From left to right:
Location
Street view
Vegetation
Ground cover

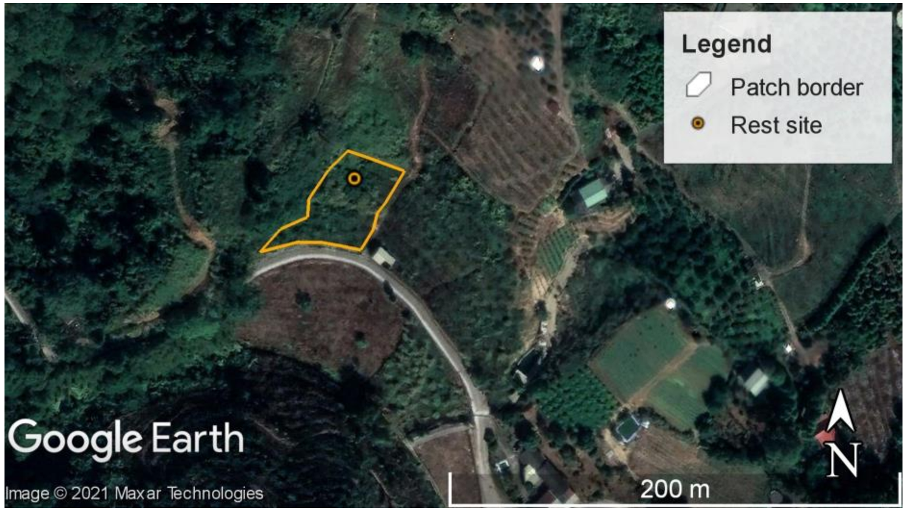

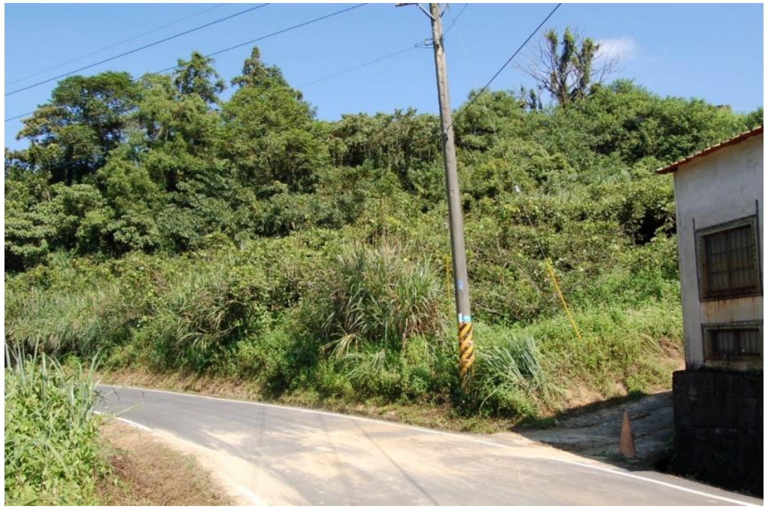
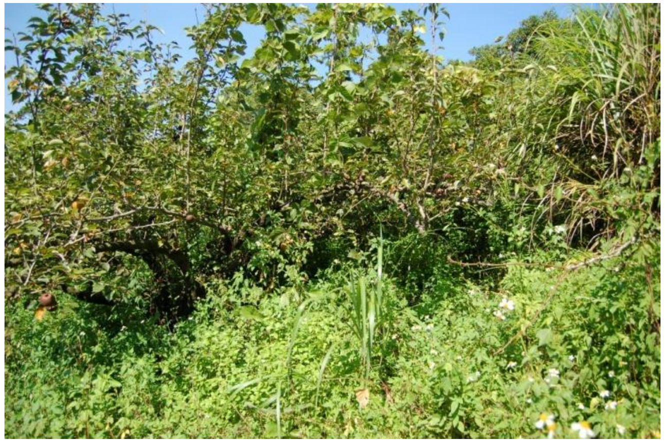
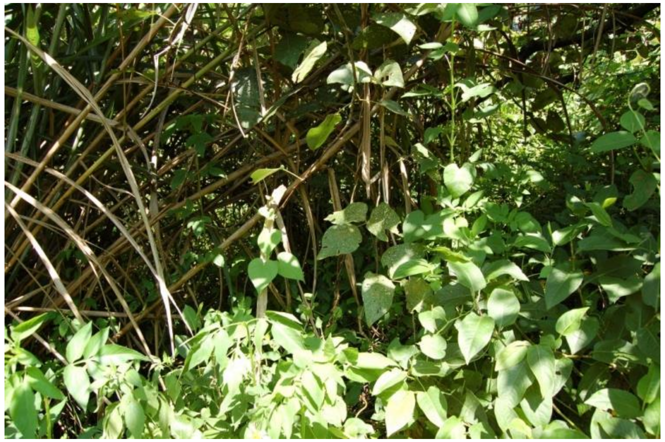

(e) Abandoned orchard

From left to right:
Location
Street view
Vegetation
Ground cover

Figure S2. Example of a semi-natural leopard cat daytime resting site consisting of concrete river blocks used by Taiwan's River Authority to manage flood water (*photo credit:* Esther van der Meer)

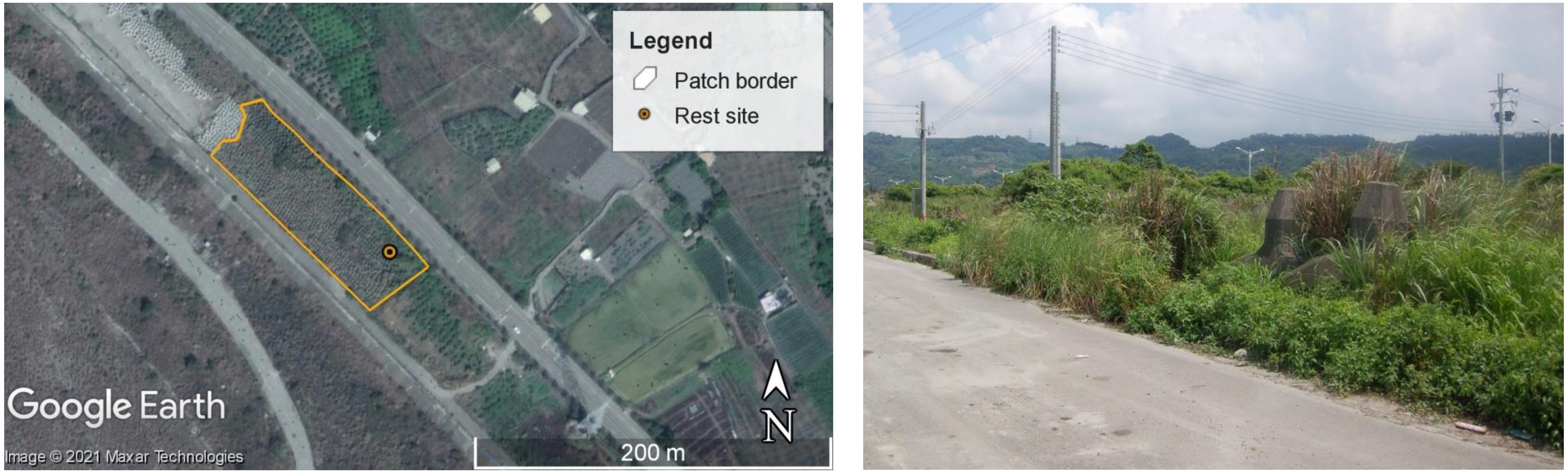

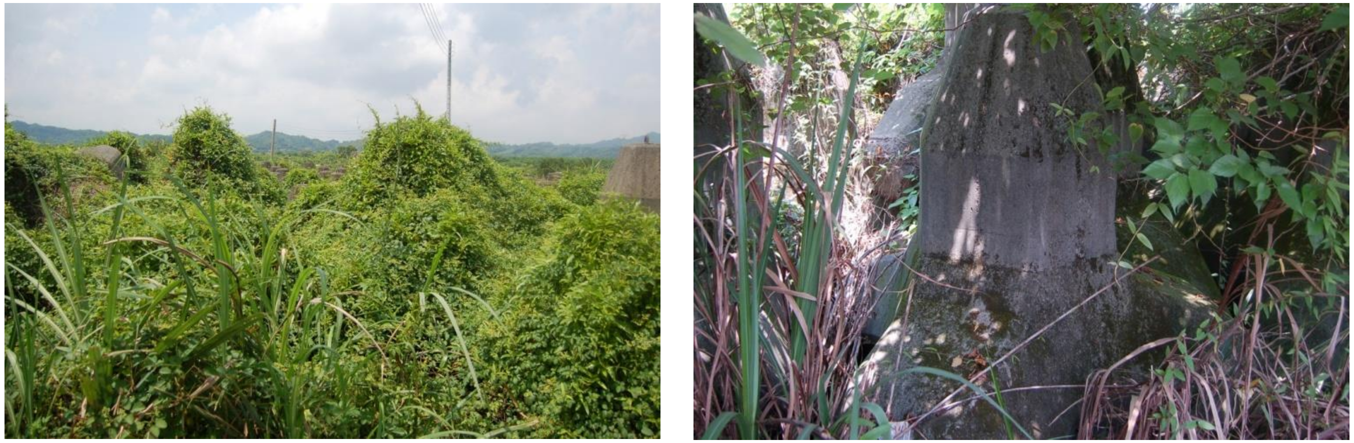

River blocks

From left to right:
Location
Street view
Vegetation
Ground cover

Figure S3. Comparison of the riverbeds for river and mountain leopard cats: the riverbed, flanked by riverine habitat, of the Da'an river which runs through the home ranges of the river cats (a) versus the riverbed of the rivers which run through the mountainous agricultural area in which the mountain cats reside (b) (*photo credit:* Esther van der Meer)

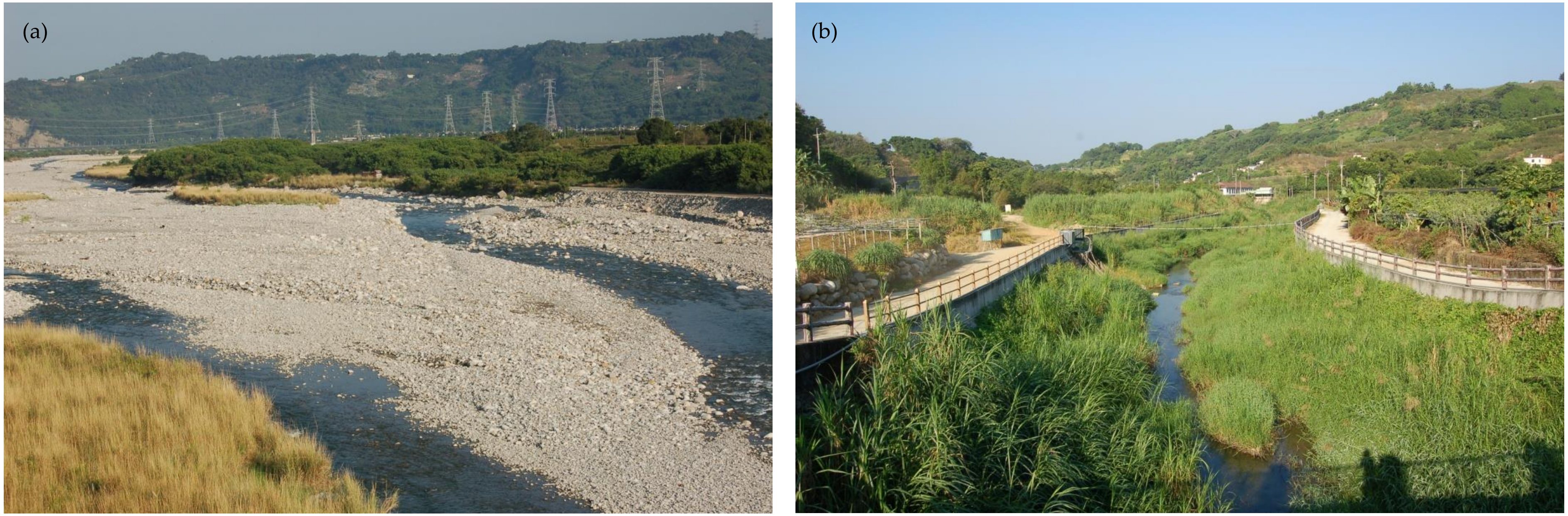

Supplement: Supplementary file 1 [file animals-13-00234-s001.zip › animals-2144803-supplementary.pdf]
